# Implementation of a tailored multifaceted antibiotic stewardship intervention to improve antibiotic prescribing for urinary tract infections in frail older adults (ImpresU) in four European countries: a process evaluation alongside a pragmatic cluster randomized controlled trial

**DOI:** 10.1186/s13063-024-08545-4

**Published:** 2024-10-18

**Authors:** Esther A. R. Hartman, Wim G. Groen, Silje Rebekka Heltveit-Olsen, Morten Lindbæk, Sigurd Høye, Sara Sofia Lithén, Pär-Daniel Sundvall, Sofia Sundvall, Egill Snaebjörnsson Arnljots, Ronny Gunnarsson, Anna Kowalczyk, Maciej Godycki-Cwirko, Alma C. van de Pol, Tamara N. Platteel, Annelie A. Monnier, Theo J. M Verheij, Cees M. P. M. Hertogh

**Affiliations:** 1 Department of Medicine for Older People, Amsterdam, UMC, Vrije Universiteit Amsterdam, De Boelelaan 1117, Amsterdam, 1081 HV The Netherlands; 2 Aging & Later Life, Amsterdam Public Health Research Institute, Amsterdam, The Netherlands; 3 Julius Center for Health Sciences and Primary Care, University Medical Center Utrecht, Utrecht University, Universiteitsweg 100, Utrecht, 3584 CG The Netherlands; 4 The Antibiotic Centre for Primary Care, Department of General Practice, Institute of Health and Society, University of Oslo, Blindern, Oslo, P.O. Box 1130, 0318 Norway; 5 General Practice/Family Medicine, School of Public Health and Community Medicine, Institute of Medicine, Sahlgrenska Academy, University of Gothenburg, Gothenburg, Box 454, 405 30 Sweden; 6 Research, Education, Development & Innovation, Primary Health Care, Region Västra Götaland, FoUUI-Centrum Södra Älvsborg, Sven Eriksonsplatsen 4, SE-503 38 Borås, Sweden; 7 Centre for Family and Community Medicine, The Faculty of Health Sciences, The Medical University of Lodz, Lodz, 90-419 Poland

**Keywords:** Urinary tract infections, Older adults, Antibiotic stewardship, Process evaluation

## Abstract

**Background:**

We previously performed a pragmatic cluster randomized controlled trial (RCT) in general practices and older adult care organizations in Poland, the Netherlands, Norway, and Sweden. We found that a multifaceted antibiotic stewardship intervention (ASI) substantially reduced antibiotic use for suspected urinary tract infections (UTIs) in frail older adults compared with usual care. We aimed to evaluate the implementation process of the ASI to provide recommendations for clinical practice.

**Methods:**

We conducted a process evaluation alongside the cluster RCT. The ASI consisted of a decision-tool and a toolbox, which were implemented using a participatory-action-research (PAR) approach with sessions for education and evaluation. We documented the implementation process of the intervention and administered a questionnaire to health care professionals (HCPs) from participating organizations in the intervention and usual care clusters. We evaluated the multiple components of the intervention and its implementation following a structured framework.

**Results:**

The questionnaire was completed by 254 HCPs from the 38 participating clusters. All components were largely delivered according to plan and evaluated as useful. The decision-tool and toolbox materials were reported to facilitate decision-making on UTIs. Regarding the PAR approach, educational sessions focusing on the distinction between UTIs and asymptomatic bacteriuria were held in all 19 intervention clusters. In 17 out of these 19 clusters, evaluation sessions took place, which were reported to help remind HCPs to implement the ASI. During both sessions, HCPs valued the reflection that took place and the resulting awareness of their behavior. It allowed them to explore implementation barriers and to tailor their local implementation process to overcome these. For example, HCPs organized extra educational sessions or revised local policies to incorporate the use of the decision-tool. Various HCPs took key roles in implementation. Staff changes and the COVID-19 pandemic were important contextual barriers.

**Conclusions:**

We found each component of the multifaceted ASI and its implementation to have added value in the process to improve antibiotic prescribing for suspected UTIs in a heterogeneous older adult care setting. We recommend using a multifaceted, multidisciplinary approach that enables HCPs to reflect on their current practice and accordingly tailor local implementation.

**Trial registration:**

ClinicalTrials.gov NCT03970356. Registered on May 31, 2019.

**Supplementary Information:**

The online version contains supplementary material available at 10.1186/s13063-024-08545-4.

## Background

Suspected urinary tract infections (UTIs) are the most common reason to prescribe antibiotics for frail older adults [1]. Often, antibiotics are inappropriately prescribed in case of non-specific symptoms such as a behavioral change or smelly urine, which are no longer considered to be indicative of UTIs [2]. Furthermore, positive urine tests frequently lead to antibiotic prescriptions, whereas this often indicates asymptomatic bacteriuria rather than a UTI [2]. To help guide physicians in their prescribing decisions, van Buul et al. developed a decision-tool for suspected UTIs in frail older adults based on consensus in an international expert panel with physicians from multiple specialties [3]. Furthermore, guidelines have incorporated these insights to encourage appropriate antibiotic prescribing for suspected UTIs [4–8]. However, a substantial behavioral change of health care professionals (HCPs) is needed to implement these principles in the older adult care setting [9].

We formed a consortium between research groups in Poland, the Netherlands, Norway, and Sweden to evaluate how to improve antibiotic prescribing for suspected UTIs in frail older adults (ImpresU) [10]. We performed a qualitative interview study through which we gained insight into the factors contributing to antibiotic prescribing for suspected UTIs in frail older adults [11]. We found that antibiotic prescriptions result from a complex decision-making process: multiple decisions by patients, caregivers, and/or nursing staff precede the physician’s prescribing decision. These decisions are influenced by the clinical situation, diagnostics, knowledge and attitudes, communication, and context and organization of care [11]. Next, we performed a pragmatic cluster randomized controlled trial, in which we evaluated the effectiveness of a multifaceted antibiotic stewardship intervention (ASI) compared to usual care in diverse care settings in general practices and older adult care organizations in the four countries. The qualitative interviews helped guide the ASI design and its implementation. In each country, the ASI was tailored based on these interviews, local guidelines, and by using readily available country-specific materials. Within clusters, the implementation could be further adjusted to accommodate for local needs and preferences in the heterogeneous older adult care setting. The trial results showed a substantial reduction in antibiotic use for suspected UTIs in frail older adults, without evidence of an increase in adverse outcomes [12].

**Fig. 1:**
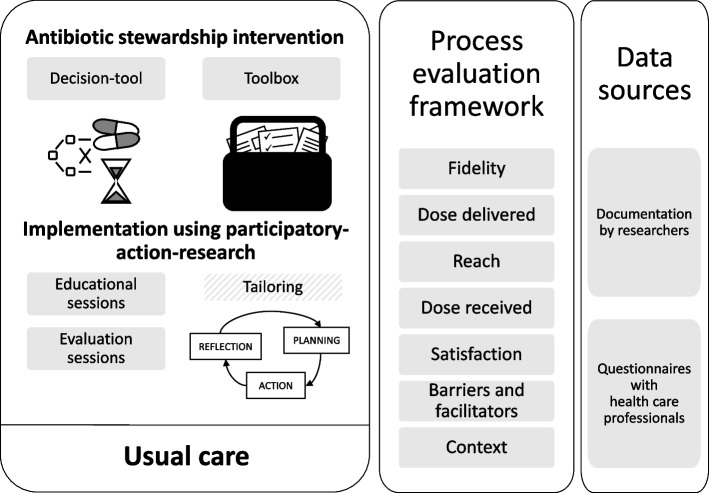
Process evaluation design. The antibiotic stewardship intervention, consisting of a decision-tool and toolbox, was implemented using a participatory-action-research approach with educational and evaluation sessions that allowed for tailoring and flexibility. We evaluated the intervention implementation and usual care, based on a structured framework, using two data sources

The ASI consisted of multiple components that were implemented using a participatory-action-research (PAR) approach to allow for tailoring to the local settings (Fig. 1). The above-mentioned decision-tool for appropriate antibiotic prescribing decisions constituted the core of the ASI (Supplemental material S1, Additional file 1) [3]. We composed a toolbox with educational materials centered around this decision-tool. These materials included pocket cards, posters, information leaflets, an active monitoring checklist, a presentation, a case study, and e-learning, targeting physicians, nursing staff, patients, and caregivers. We designed the PAR approach to include educational and evaluation sessions, in which we aimed for HCPs and researchers (together called action researchers) to complete cycles of reflection, planning, and action. We wanted to provide each cluster with at least one educational session, focusing on the distinction between UTIs and asymptomatic bacteriuria, and on use of the decision-tool. Further, we wanted each cluster to have at least one multidisciplinary evaluation session attended by all key stakeholder groups. During the sessions, we aimed for stakeholders to reflect on their current practice regarding suspected UTIs to create awareness of their own and others’ role in the process. Through PAR, we intended that stakeholders would feel actively involved in changing their practice and tailor the implementation of the ASI where needed, for example, through inclusion of different stakeholders or use of different materials.

Considering the complexity of the antibiotic prescribing decision for suspected UTIs, as well as the heterogeneity of the older adult care setting within and between countries, a detailed process evaluation is valuable [9, 11, 13]. In the cluster randomized controlled trial, we found that the ASI implementation was effective in reducing antibiotic use for suspected UTIs. This process evaluation aims to identify general lessons on how this was achieved within this diverse setting. We evaluated the implementation process of this multifaceted ASI to better understand which components contributed to it. Ultimately, we developed recommendations for further implementation across care settings for frail older adults that can be used by HCPs, antibiotic stewardship researchers, and policy makers.

## Methods

This study is part of the Improving antibiotic prescribing for frail older adults (ImpresU) project. We conducted a process evaluation alongside a cluster randomized controlled trial in general practices and older adult care organizations in Poland (PL), the Netherlands (NL), Norway (NO), and Sweden (SE), both in intervention and control clusters. Prior to this, we performed an in-depth qualitative exploration of the context of the antibiotic prescribing decision through interviews with relevant stakeholders. The protocol and results of the qualitative study and the trial (registered NCT03970356) have been published previously [10–12].

### Clusters and setting

In total, 38 clusters (PL: 8, NL: 11, NO: 9, SE: 10) participated in the trial of which 19 (PL: 4, NL: 6, NO: 4, SE: 5) received the intervention and 19 (PL: 4, NL: 5, NO: 5, SE: 5) provided care as usual. Clusters consisted of one or more general practices and/or older adult care organizations (Supplemental material S2, Additional file 1). Each general practice and older adult care organization had a local study coordinator for communication with the research team. In Poland, clusters each consisted of 1 general practice and 1 nursing home. In the Netherlands, most clusters had 1 to 3 general practices and 1 or 2 residential care homes and/or home care organizations. One Dutch cluster was an exception to this, with 10 collaborating general practices providing medical care in one residential care home. Next to general practitioners (GPs), general practice assistants and general practice nurses (position explained by Faber et al. [14]) were frequently involved in Dutch general practices. In Norway, each cluster consisted of 1 nursing home with internal nursing home physicians providing medical care. In Sweden, clusters consisted of 1 general practice and 1 or 2 nursing homes. Nurses and nurse assistants are the most important HCPs in the older adult care organizations. Further details on the setting are available in the Supplementary data of our protocol manuscript [10].

The participating care organizations varied considerably regarding the type of care, size, whether they received public or private funding, and whether they were located in a rural or urban area. For example, small-scale living facilities specific for patients with dementia were included, as well as relatively large somatic wards of nursing homes, and care organizations targeting specific groups such as veterans. Some organizations had previously participated in other research projects, while others had not.

### Process evaluation framework

We developed our process evaluation design (Fig. 1) based on the framework of Saunders et al. [15]. We used this framework to evaluate the fixed components of the ASI, i.e., the decision-tool and toolbox, and the fixed components of its implementation using PAR, i.e., the educational and evaluation sessions (Fig. 1). We assessed *fidelity*, the extent to which the components were delivered according to plan; *dose delivered*, the extent to which the components were provided by the researchers; *reach*, the extent to which HCPs were reached by the components; *dose received*, the extent to which HCPs actively used the components; and *satisfaction*, the opinion of HCPs on the components and on the ASI implementation as a whole. In the Saunders’ framework, *dose received* includes *satisfaction*. We used these terms separately to distinguish between the use of the intervention’s components by HCPs and their opinions on the components and overall on the implementation process. As it is difficult to apply this framework to the flexible aspects of the PAR approach, we also provided a description with practical examples of how action researchers tailored the implementation process. We chose not to evaluate recruitment and trial procedures as we considered this beyond the scope of evaluating the implementation of the ASI. Nevertheless, we included the perspectives of HCPs in usual care clusters because ASI implementation took place as part of a trial in a tumultuous context with the COVID-19 pandemic. Therefore, we adjusted the Saunders’ component *recruitment* and relabeled it *barriers and facilitators*, that is, for implementation of the components in intervention clusters and for general study involvement both in intervention and usual care clusters. Last, we evaluated *context*, concerning any influence of external factors both in intervention and usual care clusters.

### Data collection

The trial period was from September 2019 to June 2021. We initially planned an intervention period of 4 months (February–May 2020), in which intervention clusters would implement the ASI and control clusters would provide care as usual. Due to the COVID-19 pandemic, the study was paused in March 2020 for 6 months. In September 2020, the intervention period was resumed for a period of two more months in Poland and the Netherlands (September to October 2020) and for 3 months in Norway and Sweden (September to November 2020).

#### Intervention documentation per cluster by researchers

During the intervention period, researchers (EH, SHO, SL, PS, SS, AK, AM) documented the implementation process. They listed attendance of sessions, wrote summaries of meetings, and kept notes of communication with the participating clusters. The researchers evaluated the aspects of the Saunders’ framework through completing a structured form (Supplemental material S3, Additional file 1) for each cluster, which was entered into a database using Survalyzer software.

#### Questionnaire with health care professionals

In the last 3 months of the trial period (March to June 2021), HCPs in participating clusters were invited to complete a questionnaire with closed and open-ended questions in their local language (Supplemental material S4, Additional file 1). We pilot-tested the questionnaire with a nurse from a Dutch intervention cluster, after which several questions were adjusted. In usual care clusters, the questionnaire was short and focused on the framework aspects *barriers and facilitators* and c*ontext*. In intervention clusters, HCPs were asked to also evaluate the separate components of the ASI and its implementation. Methods for distributing the questionnaire differed across countries to minimize the burden for HCPs on top of trial procedures. In the Netherlands and Norway, HCPs completed the questionnaire online. In Poland and Sweden, HCPs completed the questionnaire on paper, and researchers entered these responses online. The researchers translated the Polish, Norwegian, and Swedish data into English. Survalyzer was used to collect these data.

### Data analysis

Quantitative data was analyzed using SPSS version 28 using descriptive statistics. For the qualitative data, we did not perform open coding because the data were already structured following the Saunders’ framework and components of the ASI implementation (Supplemental materials S3 and S4, Additional file 1). We evaluated each open-ended question separately. We categorized the responses along the themes of our previous qualitative study (clinical situation, diagnostics, knowledge and attitude, communication, and organization of care) [11]. For questions where this approach was not applicable, we categorized the responses by similarity of content. Analyses were performed by EH and re-evaluated by WG. Next, for each component of the ASI and its implementation, we wrote an extensive description of the quantitative and qualitative results. These findings were discussed within the international research team and finalized into this manuscript. Analysis took place after the results from the cluster randomized controlled trial were known.

## Results

### Characteristics of questionnaire respondents

In total, 257 HCPs completed the questionnaire, with a varying response per country (Table 1). In each cluster, a median of 5 (range 1 to 19) HCPs filled in the questionnaire, including different types of stakeholders (Table 1). Of 257 HCPs, 189 provided responses to open-ended questions (118 from intervention clusters, 71 from usual care clusters).

**Table 1:** Baseline characteristics of questionnaire respondents^a^. Values are numbers (percentages) unless otherwise stated

	Intervention (*n* = 146)	Usual care (*n* = 111)	Poland (*n* = 20)	The Netherlands (*n* = 105)	Norway (*n* = 53)	Sweden (*n* = 79)	Total (*n* = 257)
General practitioner/nursing home doctor	23 (16)	19 (17)	7 (35)	24 (23)	4 (7.5)	7 (8.8)	42 (16)
Nurse^b^	47 (32)	39 (35)	11 (55)	28 (27)	28 (53)	19 (24)	86 (34)
Nurse assistant^b^	52 (36)	44 (40)	1 (5.0)	27 (26)	16 (30)	52 (66)	96 (37)
Other profession^c^	24 (16)	9 (8.1)	1 (5.0)	26 (25)	5 (9.4)	1 (1.2)	33 (13)
Female	123 (85)	92 (83)	15 (75)	86 (82)	46 (87)	65 (82)	215 (84)
Age (median, range) in years	46 (19–73)	41 (20–75)	47 (28–73)	46 (19–66)	43 (22–62)	45 (20–75)	45 (19–75)
Working experience in their field (median, range) in years	15 (1–53)	12 (1–45)	20 (2–53)	12 (1–47)	14 (1–37)	11 (1–43)	14 (1–53)

^a^Response rate: total 257/576 (45%), Poland 20/23 (87%), Sweden 79/119 (66%), Norway 53/97 (55%), the Netherlands 105/337 (30%)

^b^All responding nurses and nurse assistants work in the older adult care organizations, except for 1 Dutch nurse and 1 Polish nurse that work in general practice

^c^Managers, head of department nurse, students, health care helpers, general practice assistants, general practice nurses

### Overall and comparative evaluation

**Fig. 2:**
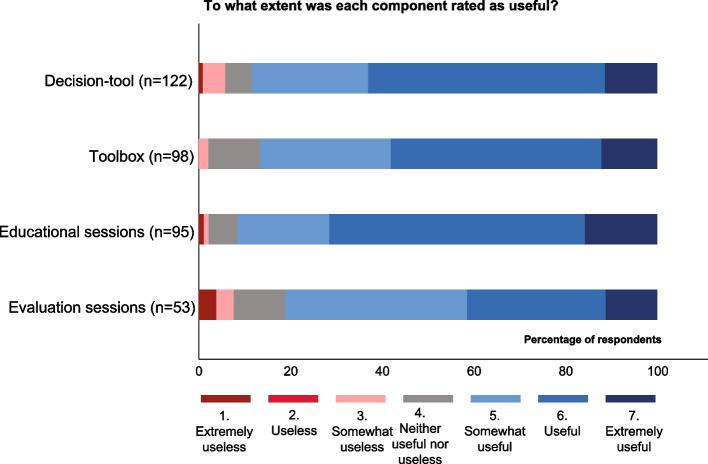
Evaluation of usefulness of components. Satisfaction of respondents with the fixed components of the antibiotic stewardship intervention and its implementation with a participatory-action-research approach

Generally, we found that all components of the ASI and its implementation with PAR were regarded as useful (Fig. 2, Table 2, Table S1 in Additional file 1). When asked what HCPs valued most, HCPs varied in their response. A Polish general practitioner described “*a bit of everything*” (Polish_GP555). It thus appears that the components separately contributed in different ways. Multiple respondents listed the increased knowledge due to the education as most valuable. Several described they had increased awareness of how to approach a UTI, with a broader perspective. Some respondents listed specific toolbox materials, while some valued the improved collaboration due to the multidisciplinary evaluation sessions. In addition, they reported to value that communication on antibiotics with informal caregivers was stimulated. Areas for improvement were listed as well. While some described that the ASI implementation could have been improved through more repetition of information or more frequent visits by the research team, others found that the study was too demanding, or that the toolbox had too many materials.

**Table 2:** Evaluation of components of the antibiotic stewardship intervention and its implementation with a participatory-action-research approach in intervention clusters. The questions regarding the sessions were only filled in by respondents who indicated to have attended the session. Results per country are provided in Table S1, Additional file 1

Question	*N* (total = 143)	Median (IQR) on a scale of 1–7
**Antibiotic stewardship intervention**		
***Decision-tool***		
1. How frequently do you use the decision-tool when confronted with UTI in frail older adults? (approximately in what % of moments you could have) 1. Never (0%) | 2. Rarely (10%) | 3. Occasionally (30%) | 4. Sometimes (50%) | 5. Frequently (70%) | 6. Usually (90%) | 7. Every time (100%)	122	4 (3–5)
2. To what extent is the decision-tool useful? 1. Extremely useless | 2. Useless | 3. Somewhat useless | 4. Neither useful nor useless | 5. Somewhat useful | 6. Useful | 7. Extremely useful	122	6 (5–6)
3. How likely is it that you would recommend the decision-tool to others? 1. Extremely unlikely | 2. Unlikely | 3. Somewhat unlikely | 4. Neither likely nor unlikely | 5. Somewhat likely | 6. Likely | 7. Extremely likely	123	6 (5–6)
***Toolbox***		
4. How many (estimated %) of your colleagues that are involved in the care around UTI in frail older adults were reached by (one or more) toolbox materials? 1. No one (0%) | 2. Hardly anyone (10%) | 3. Few of them (30%) | 4. Half of them (50%) | 5. Most of them (70%) | 6. Nearly all of them (90%) | 7. All of them (100%)	94	4 (3–6)
5. To what extent were the toolbox and its educational materials useful? Please give your opinion for each item (or choose “not applicable”) 1. Extremely useless | 2. Useless | 3. Somewhat useless | 4. Neither useful nor useless | 5. Somewhat useful | 6. Useful | 7. Extremely useful		
The toolbox in general	98	6 (5–6)
Pocket card	103	6 (6–7)
Poster	96	5 (4–6)
Information leaflet for health care professionals	68	6 (5–6)
Information leaflet for older patients and next of kin	89	5 (4–6)
E-learning/video	83	6 (5–6)
Presentation for internal education	84	6 (4.5–6)
Active monitoring checklist	76	6 (5–6)
Case study: Margareth	60	6 (5–6)
Mobile version of the decision-tool (Norway)	13	5 (5–6)
**Implementation with a participatory-action-research approach**		
***Educational sessions***		
6. To what extent was the educational session useful? If you attended more than one session, consider both in your overall opinion 1. Extremely useless | 2. Useless | 3. Somewhat useless | 4. Neither useful nor useless | 5. Somewhat useful | 6. Useful | 7. Extremely useful	95	6 (5–6)
7. The educational session(s) made us reflect on our current practices on recognition of UTIs and antibiotic use for UTIs in frail older adults 1. Strongly disagree | 2. Disagree | 3. Somewhat disagree | 4. Neither agree or disagree | 5. Somewhat agree | 6. Agree | 7. Strongly agree	98	6 (5–6)
8. The educational session(s) stimulated action to change our current practices around UTIs in frail older adults 1. Strongly disagree | 2. Disagree | 3. Somewhat disagree | 4. Neither agree or disagree | 5. Somewhat agree | 6. Agree | 7. Strongly agree	98	5 (5–6)
***Evaluation sessions***		
9. To what extent was the evaluation session useful? If you attended more than one session, consider both in your overall opinion 1. Extremely useless | 2. Useless | 3. Somewhat useless | 4. Neither useful nor useless | 5. Somewhat useful | 6. Useful | 7. Extremely useful	53	5 (5–6)
10. The evaluation session(s) made us reflect on our current practices on recognition of UTIs and antibiotic use for UTIs in frail older adults 1. Strongly disagree | 2. Disagree | 3. Somewhat disagree | 4. Neither agree or disagree | 5. Somewhat agree | 6. Agree | 7. Strongly agree	53	5 (5–6)
11. The evaluation session(s) stimulated further action to change our current practices around UTIs in frail older adults 1. Strongly disagree | 2. Disagree | 3. Somewhat disagree | 4. Neither agree or disagree | 5. Somewhat agree | 6. Agree | 7. Strongly agree	52	5 (4–6)
***Perceived changes in practice***		
12. I think that our general practice/nursing staff team improved appropriate antibiotic prescribing for UTI in frail older adults 1. Strongly disagree | 2. Disagree | 3. Somewhat disagree | 4. Neither agree or disagree | 5. Somewhat agree | 6. Agree | 7. Strongly agree	124	5 (4–6)
13. I changed my behavior when confronted with a suspicion of UTI in a frail older patient 1. Strongly disagree | 2. Disagree | 3. Somewhat disagree | 4. Neither agree or disagree | 5. Somewhat agree | 6. Agree | 7. Strongly agree	124	5.5 (4–6)
14. I feel that me and my colleagues were actively involved in changing and improving UTI care for frail older adults 1. Strongly disagree | 2. Disagree | 3. Somewhat disagree | 4. Neither agree or disagree | 5. Somewhat agree | 6. Agree | 7. Strongly agree	118	5 (4–6)
15. In our general practice/nursing staff team we made a plan and/or action points to improve UTI care for frail older adults 1. Strongly disagree | 2. Disagree | 3. Somewhat disagree | 4. Neither agree or disagree | 5. Somewhat agree | 6. Agree | 7. Strongly agree	105	4 (3–6)

### Antibiotic stewardship intervention

The decision-tool and toolbox, tailored to each country, were largely delivered according to plan. However, their reach and use in practice appeared to vary (Table 2, q1). We report detailed findings regarding *fidelity*, *dose delivered*, *reach*, and *dose received* in Supplemental material S5, Additional file 1, including an overview of toolbox materials in the four countries in Table S2. Below, we discuss *satisfaction* and *barriers and facilitators* regarding the decision-tool and toolbox.

#### Decision-tool

Generally, responding HCPs considered the decision-tool to be useful (Fig. 1, Table 2 q2). They described that the decision-tool provided a clear graphic overview, stimulated a more thorough patient evaluation, and helped to provide “*concrete observations to the doctor*” (Norwegian_nurse445). Furthermore, they reported it to provide more awareness, justification for the decision to themselves, and to facilitate explanation of the decision to colleagues, patients, and relatives. Conversely, some believed the decision-tool may come with a risk of under- or postponed treatment. Also, some found the decision-tool had too many branches. Others described the possibility of losing sight of the bigger clinical picture when focusing on using the tool.

Both in the questionnaire and during the sessions, HCPs indicated that they found it difficult to apply the decision-tool in several clinical situations with a suspected UTI. Some reported to deviate in case of a substantial symptom burden, no alternative diagnosis, a recognition of symptoms from previous UTIs, or based on gut feelings. Additionally, deviation from the decision-tool was described when assessing a patient with a suprapubic catheter and abdominal pain, or a patient with flank pain and confusion (not delirium). Recognition of specific UTI-related symptoms was described to be difficult in patients with dementia, incontinence, or diabetes mellitus. In patients with an indwelling catheter, quick deterioration was feared when antibiotics were withheld. In a Dutch session, nursing staff expressed concerns regarding the decision-tool’s advice for active monitoring in patients with confusion as the only symptom. They felt it was not patient-friendly as they perceived it as having to “wait for the delirium.”

#### Toolbox

HCPs generally rated the toolbox as useful (Fig. 2, Table 2 q5). Pocket cards displaying the decision-tool and the e-learning module were perceived as most useful (Table S1 q5, Additional file 1). Respondents described that the materials helped to grow accustomed to the new way of working and were useful in case of doubt. Conversely, some would have preferred the toolbox to be more compact “*waste of paper, too many materials makes you think pffff*” (Dutch_GP292). Some respondents reported to forget the materials when out of sight and suggested their integration into the local prescription guideline and/or electronic medical record. Respondents varied in their preferences: in multiple clusters, posters were perceived as helpful, while they were also described as unsuitable in a homely small-scale living facility for patients with dementia. Researchers perceived motivation in local study coordinators as a facilitator of good distribution of toolbox materials.

### Implementation using a participatory-action-research approach

The educational and evaluation sessions were generally delivered according to our aims, albeit with low attendance in some clusters. We report details regarding *fidelity*, *dose delivered*, and *reach* of these sessions in Supplemental material S5, Additional file 1. Here, we discuss *satisfaction* and *barriers and facilitators* regarding the sessions and provide a descriptive evaluation of the implementation process.

#### Educational sessions and evaluation sessions

Generally, the educational sessions were evaluated as “useful,” and the evaluation sessions as “somewhat useful” as displayed in Fig. 2 and Table 2 (q6, q9). To some extent, they appeared to stimulate reflection and to take action (Table 2, q7–11). Respondents described to find the educational sessions relevant and creating “*awareness of a different way of thinking and acting*” (Dutch_GP275). Some described it as an eye-opener, others as a refresher of existing knowledge. The fact that multiple disciplines received education was reported to stimulate uniform working, “*that everyone thinks the same*” (Swedish_nurse574). The ability to reflect together and exchange experiences was described as helpful. Some physicians as well as nursing staff believed the educational session was insufficiently tailored to their knowledge level. Others would have preferred a shorter session with less theory and more discussion. The evaluation sessions were described to serve as a reminder to implement and use the decision-tool. Furthermore, the evaluation session provided “*confirmation that we have started to think ‘right’*” (Swedish_nurse490). Discussing the topic with other disciplines was reported to improve the collaboration, “*in particular because the general practice assistants and the home care staff understood each other better*” (Dutch_general practice nurse282). In several clusters in the Netherlands and Norway, HCPs chose to have more than one evaluation session as further reminders to sustain motivation for implementation. In some clusters, not all key stakeholders attended the evaluation sessions (Supplemental material S5, Additional file 1). Researchers felt that the presence of specific stakeholders, such as a physician or nurse assistant, could have improved the sessions in these clusters.

During the sessions, HCPs discussed many barriers for the implementation of the decision-tool in practice and how to overcome them. The researchers recognized most barriers from the qualitative interviews [11], which helped to stimulate reflection and discussion. HCPs extensively discussed the dependence on other colleagues regarding diagnostics and decisions on suspected UTIs: for example, the dependence of GPs on nursing staff and vice versa. This indicated the need to include these colleagues in the implementation process. Knowledge gaps, habits, attitudes, and communication difficulties also came up. For example, HCPs indicated that patients and relatives often expect antibiotic treatment and thus would require more explanation when refraining from antibiotics. Logistic and organizational barriers were discussed, such as diagnostic challenges and problems out-of-hours. Several HCPs voiced the wish to incorporate the decision-tool in guidelines or integrate it into the medical record.

Multiple organizational barriers and facilitators impacted the sessions, which may explain the low attendance in some sessions. The large influence of the COVID-19 pandemic is discussed separately in Supplemental material S5, Additional file 1. Other barriers included staff shortages, planning difficulties with multiple organizations, and internal communication problems leading to the sessions not being promoted. Researchers listed mandatory attendance for sessions, the use of existing meeting structures, and good communication with the local study coordinator as organizational facilitators. Sharing quotes from the qualitative interview study was perceived to stimulate discussion. Additionally, the researchers experienced that having live sessions instead of online sessions and providing toolbox materials to HCPs in advance resulted in more interaction and reflection during sessions.

#### Tailoring implementation: practical examples

HCPs tailored their implementation to the local setting in various ways. In majority, implementation actions aimed to spread knowledge to the staff; e.g., through the internal distribution of pocket cards displaying the decision-tool. Some clusters held extra internal educational sessions; for example, by a Norwegian nursing home doctor and nurse for their staff. Some actions aimed to improve communication or organization of care. In a Polish nursing home, the use of the monitoring checklist was made mandatory. Several Dutch general practices used local “urine protocols/checklists,” which were revised to incorporate the content of the decision-tool. In one general practice, general practice assistants and GPs agreed on only discussing UTIs in older adults in front of a computer to prevent quick, potentially inappropriate decisions at the coffee table. In another general practice, the general practice nurse involved GPs from other practices to align their policies and prevent conflicting messages to the nursing staff. In a Norwegian nursing home, nursing staff agreed on using the pocket card with the decision-tool before contacting the physician for a suspected UTI. In a Swedish nursing home, nurse assistants were restricted from taking urine tests. In many clusters, additional action points were planned, but not executed. For example, Polish nursing staff planned to make an information package for new colleagues based on the toolbox, and Dutch GPs wanted to reflect on prescription data with their pharmacy.

Across clusters, different action researchers took up key roles in the implementation process. Among HCPs, nurses appeared to have the largest role. For example, they organized sessions, executed action points, and communicated between researchers and other care staff. In several clusters, physicians had an active role in implementation, e.g., by organizing internal education. The researchers contributed to implementation as well. Next to organizing the sessions, they distributed additional pocket cards in multiple clusters upon request and helped revise the local “urine checklist” in Dutch general practices. Although varying between countries, many respondents were not aware of concrete plans or action points for implementation within their cluster (Supplemental Table 2 q15). Nonetheless, respondents to some extent believed that their cluster had improved antibiotic prescribing (Table 2 q12). Multiple respondents provided examples of how they personally changed their behavior (Table 3).

**Table 3:** Responses of health care professionals with examples of how they personally changed their attitude and behavior

**Change in clinical assessment or diagnostic use**
“*I watch the patient’s symptoms carefully, I ask more.*” Polish_nurse560
“*I use the checklist to monitor resident’s symptoms.*” Polish_nurse556
“*Don’t immediately stick [urine], but more broadly watch the symptoms of the resident*.” Dutch_nurse assistant386
“*First and foremost, use preventive measures, for example, drinking enough, hygiene.*” Norwegian_nurse320
“*Be more aware of other causes if a patient is, for example, worried and goes to the toilet more often.*” Swedish_nurse assistant491
**Change in attitude when making decisions**
“*I thought about prescribing an antibiotic for a long time, and sometimes held back.*” Polish_GP558
“*Especially approach it more critically and not automatically act based on urine [test] results*” Dutch_GP244
“*More confident in my decisions.*” Swedish_nurse529
“*Dare to refrain from treatment.*” Swedish_nurse490
**Change in behavior towards colleagues**
“*We usually strip [urine] three days after the [antibiotic] course. When this came up during handover, I asked why and whether she still had complaints. She did not, it was something we just always do. It resulted in a good talk*.” Dutch_nurse374
“*Act more according to protocol and confront the GPs when they have their own theories*” Dutch_general practice nurse243

Several facilitators were reported to influence implementation. Researchers experienced many participating HCPs as intrinsically motivated; HCPs were interested in the topic and eager to improve their quality of care regarding suspected UTIs and appropriate antibiotic use. Local study coordinators were often key in motivating others and taking up responsibility within the implementation process. Awareness of the problem of antibiotic resistance was an initial motivating factor. For example, during an educational session, HCPs described their experiences in seeing the patient burden of carrying multi-resistant bacteria and requiring isolation measures. Over time, the successful application of the ASI in practice appeared as an important facilitator in the process. HCPs noticed their own progress and change in practice regarding managing suspected UTIs. For example, Norwegian HCPs experienced that next of kin was happy their relative did not need antibiotics.

### Context in both intervention and usual care clusters

#### Staff turnover and shortages

Staff turnover strongly influenced implementation and the study in general: “*the study felt like a burden[…] as we had low staffing*” (Swedish_nurse519). Respondents described the impact on implementation; for example, when new personnel had not attended the educational sessions. Changes in key persons for implementation, such as a physician, responsible nurse, head of department, or nursing home director, had a particularly large impact. In both intervention and usual care clusters, researchers experienced changes in local study coordinators as a major barrier for implementation and study procedures. Changes in local coordinators took place 41 times in 21 of 38 clusters (between 1 and 5 per cluster). Staff changes were of influence throughout the entire study period, e.g., due to pregnancy leaves or burn-out, and aggravated when the COVID-19 pandemic started.

#### COVID-19 pandemic

In intervention clusters, the COVID-19 pandemic was a major implementation barrier. The pandemic had a logistic impact on the organization of sessions. Moreover, respondents described they could not prioritize implementation due to increased workload, increased staff shortages, and “*greater stress at work and fear of infection and own health*” (Polish_nurse552). Next to hampering implementation, these problems affected the study and communication between the research team and HCPs in both intervention and control clusters. Supplemental material S5 in Additional file 1 provides further details regarding the impact of the pandemic.

#### UTI recommendations

Guidelines with UTI recommendations influenced the implementation process in intervention clusters. They may also have influenced decisions on UTIs in usual care clusters, as HCPs in usual care clusters were able to access this information. Supplemental material S5, Additional file 1 describes the local contexts and the extent of potential exposure in the usual care clusters.

## Discussion

In a pragmatic cluster randomized controlled trial, a multifaceted ASI consisting of a decision-tool and toolbox, implemented using a PAR approach within educational and evaluation sessions, was found to be effective in reducing antibiotic prescribing for suspected UTIs in frail older adults [12]. In this process evaluation, we confirm that these multiple components each have added value in the trajectory of improving antibiotic prescribing decisions. Moreover, we did not find one component to stand out in a positive or negative way. These results complement previous findings from the qualitative study and trial by providing details and concrete examples of what the implementation process entailed, and how HCPs changed their behavior [11, 12]. Based on these findings, we formulated recommendations for future implementation in clinical practice that are applicable in multiple countries (Table 4).

**Table 4:** Key recommendations for ASI implementation to improve antibiotic prescribing for suspected UTIs in frail older adults

✓Start with a local exploration: reflect on the setting and current practices when a UTI is suspected. The results from our qualitative study [11] can help guide this exploration.
✓ Use a multifaceted approach: multiple components are simultaneously needed to address the complexity of changing antibiotic prescribing decisions: ✓Consider the decision-tool as the foundation of your antibiotic stewardship program, discuss its applicability in practice with health care professionals, and address any concerns that arise. ✓Provide a toolbox with educational materials addressing all relevant stakeholders; however, ensure local selection of materials to prevent overwhelming staff through information overload. ✓Organize educational sessions, preferably live, to help overcome knowledge gaps and stimulate reflection on local practices. ✓Organize evaluation sessions with key stakeholders, preferably repeated, to stimulate multidisciplinary collaboration and to serve as a reminder to implement.
✓Use a multidisciplinary approach: identify and include all health care professionals involved in signaling, diagnostics, and antibiotic prescribing in case of suspected UTIs.
✓ Allow flexibility and adaptation of the ASI: enable health care professionals to tailor implementation to their local practice and preferences.
✓Anticipate staff turnover and staff shortages: stimulate local reflection on how to engage new staff in the ASI implementation.

A comparison with literature is difficult as process evaluation studies greatly vary in their purpose and use of methodological frameworks [15–19]. Our focus on the ASI implementation process can facilitate the transfer to application in practice. The details across the four participating European countries may provide an incentive for other countries that aim to improve antibiotic prescribing for UTIs in the older adult care setting. Similar to other studies in care settings for older adults, we found staff capacity to be an important contextual factor [20–22]. Also, many studies revealed the value of key persons for implementation or “team champions” [20, 22, 23], which is reflected in our finding of the importance of local study coordinators. We found the motivation of HCPs for the study to be an important facilitator for implementation. Likewise, an Irish qualitative study previously emphasized the importance of motivation for behavioral change in antibiotic stewardship [9].

Understanding how to achieve behavioral change has increasingly been recognized as research priority [24, 25]. It is encouraging that the participating HCPs reported to have changed their behavior (Table 3). We could not directly relate the reported behavioral changes in clusters to the trial findings, as the numbers of antibiotic prescriptions are too low for analyses at cluster-level. However, the themes identified in our prior qualitative study may help understand how the multiple components each contributed to these changes [11]. First, an antibiotic prescription is the result of consecutive decisions of multiple different HCPs. The different components simultaneously reached many stakeholders. Of these components, in particular the evaluation sessions stimulated interdisciplinary communication and collaboration. Another theme constituted the knowledge gap regarding the distinction between a UTI and asymptomatic bacteriuria, which was addressed by the decision-tool, educational sessions, and toolbox materials. Furthermore, a suspected UTI was found to trigger an “attitude to act,” resulting in a quick antibiotic prescription. The PAR element stimulated HCPs to reflect on their attitude, thereby allowing for increased awareness of their decision-making. Last, organization and context of care greatly varies and impacts antibiotic prescribing. HCPs could tailor the ASI implementation to their own preferences owing to the PAR approach.

This process evaluation has several limitations. We did not perform qualitative interviews. This could have revealed in-depth information on additional contextual factors that presumably influenced implementation, such as team dynamics and procedural drift [21]. We considered interviews not feasible due to the pandemic and collected qualitative data only through open-ended questions in the questionnaire. We pilot-tested the questionnaire with one nurse. A more rigorous validation of questionnaire design and translation would have been preferable to avoid potential misinterpretations as the questionnaire was administered to a variety of HCPs in four countries. Another limitation includes potential selection bias in the questionnaire because distribution methods and response rate substantially differed between countries. Nevertheless, we reached many different HCPs across all participating clusters. Last, an important limitation is that we did not evaluate the perspective of patients and informal caregivers.

Our findings and recommendations have several implications for policy and practice. We found that all components of the ASI and its implementation appeared to contribute in different ways. We therefore believe caution is warranted in simplifying the ASI in order to upscale its implementation. The flexibility and tailoring to local needs appeared to be key. In our study setting, this was achieved by the prior qualitative exploration and continuing exploration through PAR. This confirms the known value of local exploration and reflection, which is essential to retain in implementation activities in practice [24]. Importantly, HCPs as well as researchers invested substantial amounts of time and effort in the implementation process. In the absence of a research team, external parties contributing to implementation may thus be of value. The Swedish strategic programme against antibiotic resistance (Strama) may be an example of continuous collaboration of experts with local HCPs [26, 27]. Older adult care settings—similar to hospital settings—require structural support for sufficient human resources to perform antibiotic stewardship activities [9, 28].

## Conclusions

In this process evaluation, we found that the decision-tool, selected toolbox materials, and the PAR approach with sessions for education and evaluation together contributed to a tailored implementation process in a heterogeneous older adult care setting. We provided recommendations to guide implementation outside the study setting to improve antibiotic prescribing for suspected UTIs in frail older adults.

## Supplementary Information

Additional file 1.

## Data Availability

Supporting data is not available as the participants did not give consent for their data to be shared publicly.

## Abbreviations

ASI: Antibiotic stewardship intervention
GP: General practitioner
HCP: Health care professional
PAR: Participatory-action-research
UTI: Urinary tract infection
