# Supplementary material for: Implementation of a tailored multifaceted antibiotic stewardship intervention to improve antibiotic prescribing for urinary tract infections in frail older adults (ImpresU) in four European countries: a process evaluation alongside a pragmatic cluster randomized controlled trial
Source: Trials. 2024 Oct 18;25:691. doi: 10.1186/s13063-024-08545-4 (PMC11488242; doi:10.1186/s13063-024-08545-4)
Supplement: Supplementary file 1 — Additional file 1. [file 13063_2024_8545_MOESM1_ESM.docx]

**Implementation of a tailored multifaceted antibiotic stewardship intervention to improve antibiotic prescribing for urinary tract infections in frail older adults (ImpresU) in four European countries: a process evaluation alongside a pragmatic cluster randomized controlled trial**

**Additional File 1:**

**Supplemental material S1: Decision-tool for suspected UTIs in patients with and without catheter (1):**

**
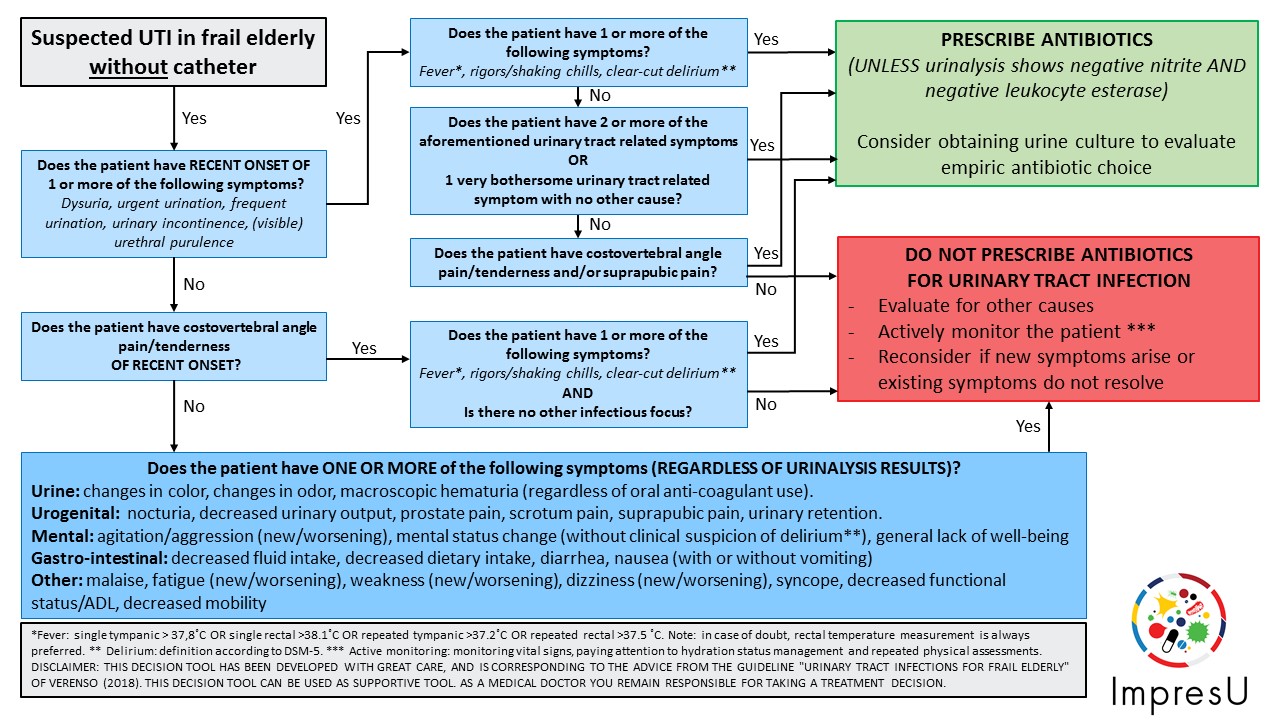

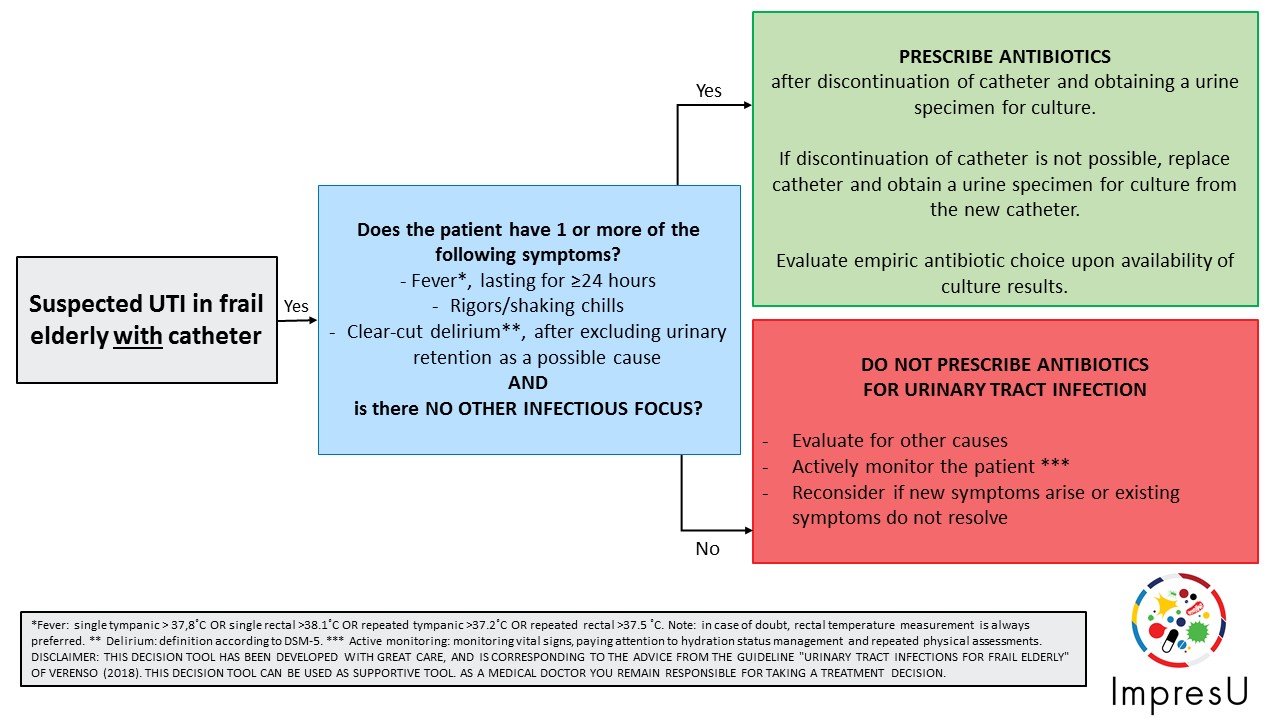
**

**Supplemental material S2: Visual description of the setting**

Clusters consisted of one or more general practices and/or older adult care organizations. Below, the setting in each country is visualized, with the health care professionals that were most frequently involved in the ASI implementation.


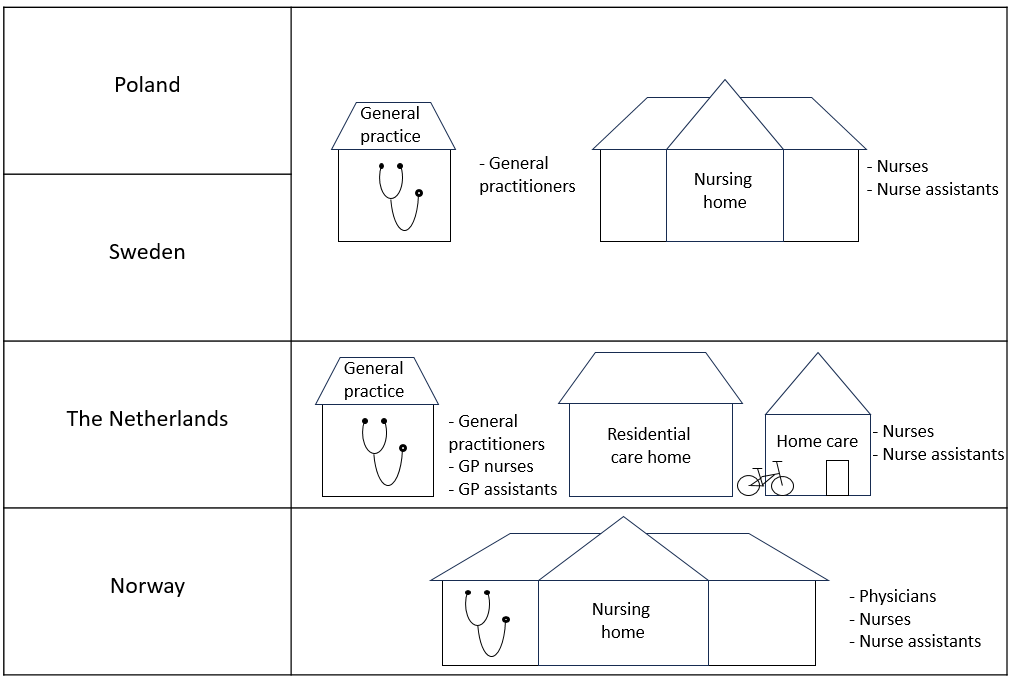


**Supplemental material S3: Structured form**

| **Structured form:** will be completed for each participating cluster, by the researchers that executed the intervention in each country, containing information on the components of the implementation of the intervention, and their perceived barriers and facilitators.  Based on sources: documents collected during the intervention period, such as summaries/minutes of meetings, attendance lists, lists of action points, e-mails, etc. | | |
| --- | --- | --- |
| *Reason to ask (Saunders)* | **Question** | **Answer (example)** |
| *Context* | Which country does the cluster belong to? | PL  NL  NO  SE |
|  | Cluster | cluster code |
|  | To which group was this cluster randomized? | Intervention  Control (🡪 skip part of the questions) |
| *Context* | Please list the type and number of organizations that the cluster consists of. | GP practice:  Nursing home:  Residential care home:  Home care organization: |
| *Context* | Please list the type and amount of stakeholders/health care professionals that are involved in the care for the cluster.  Other involved stakeholders may be managers, GP assistants, nurse practitioners, etc. | Physicians:  Nursing staff:  Other stakeholders, namely: |
| *Context* | Please describe the population within the cluster. Multiple options possible. | - Psychogeriatric - Somatic - Home care - Other, namely |
| *Context* | Please describe any other characteristics of the cluster that may be relevant.  *e.g. academic GP practice, participating ward of large nursing home, small-case dementia housing, private organization, etc.* | *Open* |
| *Context* | How many changes in contact persons for ImpresU have there been in this cluster? | N |
| *Context* | Please explain if and how changes in personnel may have influenced aspects of the study in this organization. (eg. recruitment, data collection, intervention) | Open |
| *Context* | Please explain your thoughts on any specific impact of COVID-19 on the study in this cluster, if any. | Open |
| *Context* | Please describe if and how any other external factors may have influenced the study in this cluster. | Open |
| *Recruitment* | Please describe any barriers in keeping this cluster involved in the study? | Open |
| *Recruitment* | Please describe any facilitators in keeping this cluster involved in the study? | Open |
| *Recruitment* | Are you aware of any reasons for a possible selection bias in this cluster? | Open |
| **For intervention clusters only** | | |
| ***Educational sessions:*** | | |
| *Dose delivered (completeness)* | Matrix question  Please provide details for any educational session that had to be cancelled, if applicable. | - Data - Stakeholders - Reason - Not applicable |
| Dose delivered (completeness) | How many educational sessions were held in total in this cluster? | N |
| *Dose delivered (completeness)* | Matrix question  Please provide the details for each educational session that was held.  1-6 | - Date - Duration (minutes) - Type of session: Live/Zoom - Not applicable |
|  | Please provide the attendance for each educational session in chronological order, and skip the questions on educational sessions that were not held. |  |
| *Reach*  *(participation*  *rate)* | Educational session 1:  What was the attendance?  Provide numbers for “ invited” and “ present”  If unknown how many health care professionals were invited, please put a question mark. | GPs: …  Nursing staff:….  Other, namely….  Total number: |
|  | Repeat 6 times |  |
|  | Please answer the following questions for all sessions together. Please try to be specific on stakeholder-specific factors (e.g. difficult to schedule for doctors, but it was easy for nursing staff). |  |
|  | Who were present from the research team? |  |
| *Reach*  *(participation*  *rate)* | Please list any barriers on the organization of the sessions and reaching of participants. (e.g. staff shortages) | Open |
|  | Please list any facilitators on the organization of the sessions and reaching of participants. (e.g. it was made mandatory, etc) | Open |
| *Dose received (exposure)* | The sessions made the stakeholders reflect on their current practices on recognition of UTIs and antibiotic use for UTIs in frail elderly. | 1. Strongly disagree  2. Disagree  3. Somewhat disagree  4. Neither agree or disagree  5. Somewhat agree  6. Agree  7. Strongly agree |
| *Dose received*  *(satisfaction)* | Please list any anticipated barriers that came up during the sessions on applying the intervention in practice. | Open |
| *Dose received*  *(satisfaction)* | Please list any anticipated facilitators that came up during the sessions on applying the intervention in practice. | Open |
| *Dose received*  *(satisfaction)* | Please explain what went well in the sessions. | Open |
| *Dose received*  *(satisfaction)* | Please explain how the sessions could have been improved. | Open |
|  | Any other remarks on these sessions? | Open |
| ***Evaluation sessions:***  *Same questions as for educational sessions.* | | |
| Dose delivered (completeness) | Please describe any other relevant contact moments in this cluster, if any.  e.g. phone calls or e-mails where information relevant for the process evaluation was discussed | Open |
| **Toolbox** | | |
| *Dose delivered (completeness)* | Was a toolbox provided to the cluster? If not, please explain why not. | Yes  No |
| *Reach*  *(participation*  *rate)* | Please provide your thoughts on whether all stakeholders were reached in this cluster with the toolbox, and reasons why or why not. |  |
|  | Any other remarks on the toolbox or one of the materials in this cluster? |  |
|  |  |  |
|  | **PAR component** |  |
| *Fidelity* | Please describe any tailoring of the intervention (components) in this cluster by researchers and/or local stakeholders. | Open |
| *Fidelity* | How many PAR-cycles (planning – action – reflection) took place in this cluster? |  |
| *Dose received (exposure)* | Please list specific action points that were planned AND executed, if any, in this cluster. |  |
| *Dose received (exposure)* | Please list specific action points that were planned but NOT executed, if any, in this cluster, and explain why, if known to you. |  |
| *Dose received (exposure)* | Please list which stakeholders played key roles in the execution of action points, and explain what they did. | GP ,…..  Nurse,….  Researcher, …  Other, namely… |
|  |  |  |
|  | **Final remarks** |  |
| Fidelity (quality) | Please explain if and how you think the intervention was successful in this cluster, and what contributed to this. | Open |
| Fidelity (quality) | Please explain if and how the intervention could have been improved in this cluster. | Open |

**Supplemental material S4: Questionnaire for health care professionals**

|  | **Questionnaire: for all stakeholders (GPs, nurses, nurse assistants, nurse practitioners, GP assistants, others)**  *By filling in this questionnaire, you will help us to better understand the results of the ImpresU study, and to understand what factors may impact UTI care in frail elderly. Thank you!* | |
| --- | --- | --- |
| *Reason to ask (Saunders)* | **For both intervention and control clusters** | |
|  | **Question** | **Answer** |
|  | **General information**  *Please fill in some general information about yourself and your organization.* | |
| *Context* | Which cluster do you work in? | Amsterdam / Loosdrecht / etc |
|  | In what organization do you work? | GP practice  Nursing home  Residential care home  Home care organization |
|  | What is your profession? | GP / nursing home doctor  Nurse  Nurse assistant  Other, namely |
|  | What is your age? | …. years |
|  | What is your gender? | Male/ Female /Other |
|  | How many years of working experience do you have in your field? | …. years |
| *Context* | How frequently are you approximately confronted with a UTI suspicion in a frail elderly patient in your daily practice? | 1. Never 2. Rarely (once or twice a year) 3. Occasionally (every few months) 4. Sometimes (monthly) 5. Frequently (twice a month) 6. Often (weekly) 7. All the time (daily) |
|  | **Questions only for intervention clusters**  *The aim of the study is to improve the recognition of UTI and appropriate antibiotic prescribing for UTI in frail elderly. In order to do this, your GP practice/nursing staff team has received an intervention. Central in this, is a decision tool. Also, a toolbox with educational materials was provided to help apply this in daily practices. Furthermore, educational sessions and evaluation sessions were organized. In the whole process, the researchers tried to actively involve you and your colleagues, to help you make any changes in a way that is best suited for your GP practice/nursing staff team.*  *The following questions are about the separate parts of the intervention.* | |
|  | **Decision tool**  *In this study, a decision tool was introduced to assist in decision-making on UTI in frail eldery, based on the symptoms that the patient presents with.* | |
| *Dose received (exposure)* | Are you familiar with the decision tool?  *add pictures!* | Yes  Somewhat  No 🡪 skip following questions |
| *Reach (participation rate)* | How frequently do you use the decision tool when confronted with UTI in frail elderly? (approximately in what % of moments you could have) | 1. Never (0%) 2. Rarely (10%) 3. Occasionally (30%) 4. Sometimes (50%) 5. Frequently (70%) 6. Usually (90%) 7. Every time (100%) |
| *Dose received (satisfaction)* | To what extent is the decision tool useful? | 1. Extremely useless 2. Useless 3. Slightly useless 4. Neither useful nor useless 5. Slightly useful 6. Useful 7. Extremely useful |
|  | How likely is it that you would you recommend the decision tool to others? | 1. Extremely unlikely 2. Unlikely 3. Slightly unlikely 4. Neither likely nor unlikely 5. Slightly likely 6. Likely 7. Extremely likely |
| *Reach (participation rate)* | What are advantages, if any, of using the decision tool? | Open |
|  | What are difficulties, if any, in using the decision tool? Do you have any suggestions for improvement? | Open |
|  | If you deviated from the advice of the decision tool, what were the reasons for that? | Open |
|  | **Educational sessions**  *In most participating organizations, educational sessions about UTI in frail elderly were held, in Feb/March 2020 and after restart of the study, between September and December 2020.* | |
| *Reach (participation rate)* | Did you attend an educational session for your GP practice / nursing staff team? | Yes, in Feb/March 2020  Yes, between September / December 2020  Yes, in both time periods  No 🡪 skip following questions |
| *Dose received*  *(satisfaction)* | To what extent was the educational session useful?  If you attended more than one session, consider both in your overall opinion. | 1. Extremely useless 2. Useless 3. Slightly useless 4. Neither useful nor useless 5. Slightly useful 6. Useful 7. Extremely useful |
| *Fidelity (quality) / Dose received (exposure)* | The session(s) made us reflect on our current practices on recognition of UTIs and antibiotic use for UTIs in frail elderly. | 1. Strongly disagree  2. Disagree  3. Somewhat disagree  4. Neither agree or disagree  5. Somewhat agree  6. Agree  7. Strongly agree |
|  | The session(s) stimulated action to change our current practices around UTIs in frail elderly. | 1. Strongly disagree  2. Disagree  3. Somewhat disagree  4. Neither agree or disagree  5. Somewhat agree  6. Agree  7. Strongly agree |
| *Dose received*  *(satisfaction)* | Please provide feedback on how the educational session was useful. | Open |
|  | Please provide feedback on how the educational session could be improved. | Open |
|  | **Evaluation sessions**  *In most participating organizations, sessions were held to evaluate.* | |
| *Reach (participation rate)* | Did you attend an evaluation session in your organization? | Yes  No 🡪 skip following questions |
| *Dose received*  *(satisfaction)* | To what extent was the evaluation session useful?  If you attended more than one session, consider both. | 1. Extremely useless 2. Useless 3. Slightly useless 4. Neither useful nor useless 5. Slightly useful 6. Useful 7. Extremely useful |
| *Fidelity (quality) / Dose received (exposure)* | The session(s) made us reflect on our current practices on recognition of UTIs and antibiotic use for UTIs in frail elderly. | 1. Strongly disagree  2. Disagree  3. Somewhat disagree  4. Neither agree or disagree  5. Somewhat agree  6. Agree  7. Strongly agree |
|  | The session(s) stimulated further action to change our current practices around UTIs in frail elderly. | 1. Strongly disagree  2. Disagree  3. Somewhat disagree  4. Neither agree or disagree  5. Somewhat agree  6. Agree  7. Strongly agree |
| *Dose received*  *(satisfaction)* | Please provide feedback on how the evaluation session was useful. | Open |
|  | Please provide feedback on how the evaluation session could be improved. | Open |
|  | **Toolbox**  *In most participating organizations, a toolbox with educational materials was provided to the organization to improve the care for UTI in frail elderly. In the organization, it was decided which materials to use, and sometimes to adapt materials to the organization. Examples of materials are an e-learning, pocket cards and posters.* | |
| *Dose received*  *(exposure + satisfaction)* | To what extent were the toolbox and its educational materials useful? Please give your opinion for each item (or click “not applicable”).   - The toolbox in general - Pocket card - Poster - Information leaflet for myself - Information leaflet for patients - E-learning / video - Powerpoint presentation for internal education - Active monitoring checklist - Case study: Margareth - Mobile version of the decision tool | 1. Extremely useless 2. Somewhat useless 3. Slightly useless 4. Neither useful nor useless 5. Slightly useful 6. Somewhat useful 7. Extremely useful   Not applicable |
| *Fidelity (quality)* | Are you aware of other materials that are used in your organization to improve the care for frail elderly with UTI? | Yes, namely..  No |
| *Reach*  *(participation*  *rate)* | How many (estimated %) of your colleagues that are involved in the care around UTI in frail elderly were reached by (one or more) toolbox materials? | 1. No one (0%) 2. Hardly anyone (10%) 3. Few of them (30%) 4. Half of them (50%) 5. Most of them (70%) 6. Nearly all of them (90%) 7. All of them (100%)   I don’t know |
| *Dose received*  *(satisfaction)* | Please provide feedback on how the toolbox and its materials were useful. | Open |
|  | Please provide feedback on how the toolbox and its materials could be improved. | Open |
|  | **Changes in care for frail elderly with UTI**  *In the process of changing and improving the care for frail elderly with UTI, the researchers tried to actively involve you and your colleagues, to help you make any changes in a way that is best suited for your organization.*  *Please answer the following questions on how study participation may have changed the care around UTI for frail elderly in your GP practice/nursing staff team.*  *To what extent do you agree with the following statements?* | |
| *Dose received (exposure)*  *Fidelity (quality)* | I think that our GP practice/nursing staff team improved appropriate antibiotic prescribing for UTI in frail elderly. | 1. Strongly disagree  2. Disagree  3. Somewhat disagree  4. Neither agree or disagree  5. Somewhat agree  6. Agree  7. Strongly agree |
| *Fidelity (quality)* | I changed my behavior when confronted with a suspicion of UTI in a frail elderly patient. | 1. Strongly disagree  2. Disagree  3. Somewhat disagree  4. Neither agree or disagree  5. Somewhat agree  6. Agree  7. Strongly agree |
|  | If possible, please give an example of how you changed your behavior. | *Open* |
| *Dose received (exposure)*  *Fidelity (quality)* | I feel that me and my colleagues were actively involved in changing and improving UTI care for frail elderly. | 1. Strongly disagree  2. Disagree  3. Somewhat disagree  4. Neither agree or disagree  5. Somewhat agree  6. Agree  7. Strongly agree I don’t know |
| *Dose received (exposure)* | In our GP practice/nursing staff team we made a plan and/or action points to improve UTI care for frail elderly. | 1. Strongly disagree  2. Disagree  3. Somewhat disagree  4. Neither agree or disagree  5. Somewhat agree  6. Agree  7. Strongly agree I don’t know |
| *Dose received (exposure)* | Please list any action(s) that you are aware of that have been taken to improve UTI care in your GP practice/nursing staff team. | Open  I don’t know |
| *Dose received*  *(satisfaction)* | Which action(s) were most helpful, and why? | Open  I don’t know |
| *Dose received*  *(satisfaction)* | Please provide feedback on how the plan and/or actions could have been improved. | Open  I don’t know |
| **For both intervention and control clusters** | | |
|  | **Context**  *Changes within your organization and external influences (such as the COVID-19 pandemic) may have impacted UTI care and study involvement of your organization.* | **Also for control group** |
| *Context* | Are you aware of the existence of a guideline concerning UTI in frail elderly? | Yes, namely  No 🡪 skip next question |
|  | How frequently do you use this guideline when confronted with UTI in frail elderly? (in what % of moments you could have) | 1. Never (0%) 2. Rarely (10%) 3. Occasionally (30%) 4. Sometimes (50%) 5. Frequently (70%) 6. Usually (90%) 7. Every time (100%) |
| *Context* | Did you or your GP practice/nursing staff team take part in any (not ImpresU related) (online) meetings / trainings / projects involving UTI care and/or use of antibiotics? | Yes, namely  No |
| *Context* | Do you think there has been a change in antibiotic prescribing for UTI in frail elderly in your GP practice/nursing staff team during the study period? | Yes, **less** antibiotic prescribing  Yes, **more** antibiotic prescribing  No change |
| *Context* | How many changes of personnel (staff turnover) are approximately happening in your GP practice/nursing staff team?  It is possible to comment on your answer below. | 1. None at all 2. Hardly any 3. Few 4. Some 5. Frequent 6. Many 7. Extremely many   I don’t know |
|  | If applicable, please explain if and how changes of personnel may have influenced UTI care within your GP practice/nursing staff team. | Open |
| *Context* | Please explain how the COVID-19 pandemic may have influenced UTI care in your organization? Multiple answers are possible. | Less direct patient contact  Less urine testing  Symptoms are attributed to possible COVID-19 instead of UTI  Less antibiotics for UTI  More antibiotics for UTI  No large impact  Other, namely… |
| *Context* | Please list and explain any other factors (e.g. organizational change) that may have impacted UTI care within your organization. | Open |
| *Context* | Please list and explain any factors that may have impacted **study participation** (e.g. recruitment, UTI registration) of your organization. | Open |
| *Dose received*  *(satisfaction)* | Please provide feedback on what was useful about study participation. | Open |
| *Dose received*  *(satisfaction)* | Please provide feedback on how we could have improved study participation in your organization. | Open |
|  | Any other comments on the study or on this questionnaire? | Open |

| **Supplemental Table S1: Evaluation of intervention components in intervention clusters with separate results for each country (Total N = 143)** | | | | | | |
| --- | --- | --- | --- | --- | --- | --- |
|  |  | **Median (IQR) on a scale of 1-7** | | | | |
| **Question** | **Total N**  **(N per country)** | **Total** | **Poland  (PL)** | **The Netherlands (NL)** | **Norway  (NO)** | **Sweden (SE)** |
| **Antibiotic stewardship intervention** |  |  |  |  |  |  |
| **Decision tool** |  |  |  |  |  |  |
| 1. How frequently do you use the decision tool when confronted with UTI in frail older adults? (approximately in what % of moments you could have)  1. Never (0%) \| 2. Rarely (10%) \| 3. Occasionally (30%) \| 4. Sometimes (50%) \| 5. Frequently (70%) \| 6. Usually (90%) \| 7. Every time (100%) | 122  (PL 11, NL 58, NO 18, SE 35) | 4 (3-5) | 4 (3-4) | 4 (3-5) | 4 (3-5) | 4 (2-5) |
| 2. To what extent is the decision tool useful?  1. Extremely useless \| 2. Useless \| 3. Slightly useless \| 4. Neither useful nor useless \| 5. Slightly useful \| 6. Useful \| 7. Extremely useful | 122  (PL 11, NL 58, NO 18, SE 35) | 6 (5-6) | 5 (4-6) | 6 (5-6) | 6 (6-6) | 5 (5-6) |
| 3. How likely is it that you would you recommend the decision tool to others?  1. Extremely unlikely \| 2. Unlikely \| 3. Slightly unlikely \| 4. Neither likely nor unlikely \| 5. Slightly likely \| 6. Likely \| 7. Extremely likely | 123  (PL 11, NL 58, NO 18, SE 36) | 6 (5-6) | 5 (4-5) | 6 (5-6) | 6 (6-7) | 5 (4-7) |
| **Toolbox** |  |  |  |  |  |  |
| 4. How many (estimated %) of your colleagues that are involved in the care around UTI in frail older adults were reached by (one or more) toolbox materials?  1. No one (0%) 2. Hardly anyone (10%) 3. Few of them (30%) 4. Half of them (50%) 5. Most of them (70%) 6. Nearly all of them (90%) 7. All of them (100%) | 94  (PL 10, NL 47, NO 15, SE 22) | 4 (3-6) | 3 (1-6) | 4 (3-6) | 4 (4-6) | 4 (3-6) |
| 5. To what extent were the toolbox and its educational materials useful? Please give your opinion for each item (or click “not applicable”).  1. Extremely useless \| 2. Useless \| 3. Slightly useless \| 4. Neither useful nor useless \| 5. Slightly useful \| 6. Useful \| 7. Extremely useful |  |  |  |  |  |  |
| The toolbox in general | 98  (PL 10, NL 43, NO 16, SE 29) | 6 (5-6) | 6 (5-6) | 6 (5-6) | 6 (5-7) | 6 (5-6) |
| Pocket card | 103  (PL 10, NL 46, NO 15, SE 32) | 6 (6-7) | 6 (5-6) | 6 (6-6) | 6 (6-6) | 6 (6-7) |
| Poster | 96  (PL 10, NL 42, NO 16, SE 28) | 5 (4-6) | 5 (4-5) | 5 (4-6) | 6 (5-7) | 6 (5-7) |
| Information leaflet for health care professionals | 68  (PL 10, NL 42, NO 16) | 6 (5-6) | 5 (4-6) | 6 (5-6) | 6 (5-6) | n/a |
| Information leaflet for patients | 89  (PL 10, NL 36, NO 15, SE 28) | 5 (4-6) | 3 (2-4) | 5 (4-6) | 6 (5-7) | 6 (5-7) |
| E-learning in The Netherlands and Norway / Video in Sweden | 83  (NL 44, NO 13, SE 26) | 6 (5-6) | n/a | 6 (6-6) | 6 (6-6) | 5 (5-6) |
| Powerpoint presentation for internal education | 84  (PL 10, NL 36, NO 14, SE 24) | 6 (4.5-6) | 4 (4-5) | 6 (6-6) | 6 (4-6) | 6 (4-7) |
| Active monitoring checklist | 76  (PL 8, NL 34, NO 14, SE 20) | 6 (5-6) | 6 (5-6) | 6 (5-6) | 6 (4-6) | 6 (4-7) |
| Case study | 60  (NL 27, NO 14, SE 19) | 6 (5-6) | n/a | 6 (5-6) | 5 (4-6) | 6 (4-6) |
| Mobile version of the decision tool (Norway) | 13  (NO) | 5 (5-6) | n/a | n/a | 5 (5-6) | n/a |
| **Participatory-action-research approach** |  |  |  |  |  |  |
| **Educational sessions** |  |  |  |  |  |  |
| 6. To what extent was the educational session useful?  If you attended more than one session, consider both in your overall opinion.  1. Extremely useless \| 2. Useless \| 3. Slightly useless \| 4. Neither useful nor useless \| 5. Slightly useful \| 6. Useful \| 7. Extremely useful | 95  (PL 11, NL 42, NO 11, SE 31) | 6 (5-6) | 6 (5-6) | 6 (6-6) | 6 (6-6) | 6 (5-7) |
| 7. The session(s) made us reflect on our current practices on recognition of UTIs and antibiotic use for UTIs in frail older adults.  1. Strongly disagree \| 2. Disagree \| 3. Somewhat disagree \| 4. Neither agree or disagree \| 5. Somewhat agree \| 6. Agree \| 7. Strongly agree | 98  (PL 11, NL 43, NO 12, SE 32) | 6 (5-6) | 5 (5-6) | 6 (5-6) | 6 (5-6) | 6 (5-7) |
| 8. The session(s) stimulated action to change our current practices around UTIs in frail older adults.  1. Strongly disagree \| 2. Disagree \| 3. Somewhat disagree \| 4. Neither agree or disagree \| 5. Somewhat agree \| 6. Agree \| 7. Strongly agree | 98  (PL 11, NL 43, NO 12, SE 32) | 5 (5-6) | 5 (4-5) | 6 (5-6) | 6 (3-6) | 6 (4-7) |
| **Evaluation sessions** |  |  |  |  |  |  |
| 9. To what extent was the evaluation session useful?  If you attended more than one session, consider both.  1. Extremely useless \| 2. Useless \| 3. Slightly useless \| 4. Neither useful nor useless \| 5. Slightly useful \| 6. Useful \| 7. Extremely useful | 53  (PL 10, NL 16, NO 10, SE 17) | 5 (5-6) | 5 (5-5) | 6 (5-6) | 6 (5-6) | 5 (4-6) |
| 10. The session(s) made us reflect on our current practices on recognition of UTIs and antibiotic use for UTIs in frail older adults.  1. Strongly disagree \| 2. Disagree \| 3. Somewhat disagree \| 4. Neither agree or disagree \| 5. Somewhat agree \| 6. Agree \| 7. Strongly agree | 53  (PL 10, NL 16, NO 10, SE 17) | 5 (5-6) | 5 (4-5) | 6 (5-6) | 6 (5-6) | 6 (5-7) |
| 11. The session(s) stimulated further action to change our current practices around UTIs in frail older adults.  1. Strongly disagree \| 2. Disagree \| 3. Somewhat disagree \| 4. Neither agree or disagree \| 5. Somewhat agree \| 6. Agree \| 7. Strongly agree | 52  (PL 10, NL 16, NO 10, SE 16) | 5 (4-6) | 4 (3-5) | 5 (5-6) | 5 (4-6) | 6 (4-7) |
| **In practice** |  |  |  |  |  |  |
| 12. I think that our general practice/nursing staff team improved appropriate antibiotic prescribing for UTI in frail older adults.  1. Strongly disagree \| 2. Disagree \| 3. Somewhat disagree \| 4. Neither agree or disagree \| 5. Somewhat agree \| 6. Agree \| 7. Strongly agree | 124  (PL 10, NL 60, NO 16, SE 38) | 5 (4-6) | 5 (4-5) | 5 (4-6) | 6 (4-7) | 5 (4-6) |
| 13. I changed my behaviour when confronted with a suspicion of UTI in a frail older patient.  1. Strongly disagree \| 2. Disagree \| 3. Somewhat disagree \| 4. Neither agree or disagree \| 5. Somewhat agree \| 6. Agree \| 7. Strongly agree | 124  (PL 10, NL 60, NO 16, SE 38) | 5.5 (4-6) | 5 (5-5) | 6 (5-6) | 5 (4-6) | 5 (4-7) |
| 14. I feel that me and my colleagues were actively involved in changing and improving UTI care for frail older adults.  1. Strongly disagree \| 2. Disagree \| 3. Somewhat disagree \| 4. Neither agree or disagree \| 5. Somewhat agree \| 6. Agree \| 7. Strongly agree | 118  (PL 10, NL 58, NO 16, SE 34) | 5 (4-6) | 5 (4-5) | 5 (4-6) | 5 (5-7) | 5 (4-6) |
| 15. In our general practice/nursing staff team we made a plan and/or action points to improve UTI care for frail older adults.  1. Strongly disagree \| 2. Disagree \| 3. Somewhat disagree \| 4. Neither agree or disagree \| 5. Somewhat agree \| 6. Agree \| 7. Strongly agree | 105  (PL 10, NL 51, NO 14, SE 30) | 4 (3-6) | 2 (2-3) | 5 (4-6) | 6 (4-6) | 4 (4-6) |

**Supplemental material S5: Additional results**

**Antibiotic stewardship intervention**

***Decision tool*** *(fidelity, dose delivered, reach, dose received)*

The researchers provided the decision-tool to each of the intervention clusters. The majority of respondents reported to be familiar (n=94/143, 66%) or somewhat familiar (n=36/143, 25%) with the decision tool. All 22 responding physicians considered themselves familiar (n=18/22, 82%) or somewhat familiar (n=4/22, 18%) with the decision-tool. Among responding nursing staff, 60/97 (62%) considered themselves familiar, and 27/97 (28%) somewhat familiar. However, respondents – both physicians and nursing staff - estimated to use the decision-tool in half of the cases of suspected UTIs in clinical practice (Table 2 q1).

***Toolbox*** *(fidelity, dose delivered, reach, dose received)*

The toolbox was provided to each cluster. Although difficult to measure, further spread of toolbox materials within clusters appeared suboptimal: respondents described that ‘half’ of their colleagues were reached by one or more toolbox materials in the Netherlands, Norway, and Sweden, and ‘few’ in Poland (Table S1 q4). Some respondents indeed described not to have seen the toolbox at all, and several described it was not used a lot: ‘*The toolkit is good, the only problem is using it’, Polish_nurse488.*

Researchers tailored the toolbox to each country and added locally available materials, for example from guidelines (overview in table S2). In Poland, the toolbox was relatively compact considering previous experience and the absence of existing local materials. Interestingly, the monitoring checklist was described as actively used in Poland whereas it appeared to receive little attention in the other countries. In the Netherlands, the qualitative interviews revealed a pivotal role for general practice assistants (2); the researchers thus added materials to the toolbox that targeted them. Within multiple Dutch clusters, the e-learning module intended for nursing staff was also used by general practice assistants. In Norway, researchers added a mobile link to the decision-tool as suggested in the interviews. The Norwegian toolbox additionally included a report with local antibiotic use, as also used in the local “RASK” intervention (3). In two Norwegian nursing homes, HCPs used a locally designed UTI screening form and checklist. In another, an externally available e-learning module was used (4). In a Swedish nursing home, the toolbox was placed on the local intranet, and a video for residents and relatives was presented at their external internet page.

**Implementation using a participatory-action-research approach**

***Educational sessions*** *(fidelity, dose delivered, reach)*

Each of the 19 intervention clusters received between 1 and 6 educational sessions (48 in total), which includes refresher sessions after the COVID-19 pause. No educational sessions took place in one general practice and one residential care home (part of two separate) Dutch clusters. These facilities no longer wished to participate in the intervention after the COVID-19 pause. In Poland and Norway, sessions were multidisciplinary for both physicians and nursing staff. In the Netherlands and Sweden, separate sessions were held for GPs and nursing staff. Nursing staff included specialized nurses, nurses, nurse assistants, and medical caregivers. A variety of other professionals attended, including managers, general practice nurses, general practice assistants, medical students, nurse practitioners. The sessions were provided by 1 to 3 researchers (PL: AK, MGC, dr. Katarzyna Kosiek, NL: EH, WG, AM, TV, CH, NO: SHO, ML, SL, SE: PS, SS, ESA), and median 5 (range 1 to 21) participants attended. The sessions had a median duration of 60 minutes (range 40 to 120), and the majority (30 of 48) were online due to the pandemic.

***Evaluation sessions*** *(fidelity, dose delivered, reach)*

In total 25 evaluation sessions were held in 17 of 19 intervention clusters (1 to 3 per cluster). The median duration was 30 (range 30-60) minutes, and again the majority (22/25) were held online. As these sessions included only ‘key’ stakeholders in the cluster, the attendance was relatively low with median 3 HCPs (range 1 to 16), and 1 to 3 researchers (PL: AK, dr. Katarzyna Kosiek, NL: EH, NO: SHO, ML, SL, SE: PS, SS). Most sessions were multidisciplinary, and attendants included GPs, nursing staff, medical caregivers, department managers, general practice nurses (NL) and general practice assistants (NL). In Norway and Sweden, physicians were not present at these meetings due to illness or other priorities.

**Context in both intervention and usual care clusters**

***COVID-19 pandemic***

The COVID-19 pandemic substantially impacted implementation in intervention clusters. At the start of the pandemic in March 2020, 10 educational sessions were cancelled and had to be rescheduled. In all countries, HCPs were unaccustomed to digital meetings leading to technical difficulties. Social distancing affected the attendance due to a lack of space to accommodate all participants. Both HCPs and researchers reported to prefer live educational sessions over online meetings as they experienced more interaction and reflection in live sessions. However, regarding the later evaluation sessions, meeting online was described as useful because participants had become accustomed to it by then. Meeting online simplified planning, and participants could join even while quarantined. Importantly, HCPs described the pandemic negatively impacted implementation due to increased workload. For example, older adults had increased care needs, and HCPs had additional tasks, such as the organization of vaccination rounds. Moreover, the pandemic impacted staffing. Sick and quarantined personnel caused staff shortages, and HCPs were often relocated across wards.

Study procedures and communication between the research team and the HCPs were also affected by the pandemic. For example, restrictions impeded researchers to visit the care facilities. Furthermore, many clusters had COVID-19 outbreaks, during which there usually was no contact between researchers and HCPs for several weeks.

***UTI recommendations in usual care***

In Poland, a guideline regarding UTIs is available which is not specific to frail older adults but addresses asymptomatic bacteriuria (5). No relevant antibiotic stewardship interventions were available in Poland. In the Netherlands, a guideline for elderly care physicians is available specific to UTIs in older adults, including the decision-tool (6). Dutch GPs mostly use GP guidelines; however, nursing staff in care homes may have been exposed to this guideline through collaborating with elderly care physicians. Additionally, a revised UTI guideline for GPs was released during the intervention period which is in line with our ASI but not specific to frail older adults (7). In the Netherlands, relevant antibiotic stewardship activities were available but mostly targeted nursing homes, which did not participate in the study. In Norway, a UTI guideline is available including recommendations specific for frail older adults (8). Additionally, the “Noklus” offers relevant educational materials and the “RASK” antibiotic stewardship program has been active in Norway but this was not yet implemented in the nursing homes participating in the study (3, 4). In Sweden, guidelines include recommendations specific for frail older adults (9). Moreover, the Swedish strategic programme against antibiotic resistance (Strama) has extensively given attention to this topic since several years (10).

Across all countries, 41/92 (45%) of responding HCPs in the usual care group (13/17 (77%) of physicians) reported to know above-mentioned guidelines concerning UTIs in older adults. Also, 15/92 (16%) HCPs in the usual care group described to have participated in UTI-related activities during the study, which included regional training and Strama activities. Last, HCPs indicated that registering UTIs in the study increased their awareness of their management of suspected UTIs.

| **Supplemental table S2** | | | |
| --- | --- | --- | --- |
| **Toolbox content in each country** | | | |
| **Poland** | **Netherlands** | **Norway** | **Sweden** |
| Pocket cards (physicians, nursing staff) | Pocket cards ( physicians, general practice assistants, nursing staff) | Pocket cards (physicians, nursing staff) | Pocket cards (physicians, nursing staff) |
| Posters (nursing staff, patients/next of kin) | Posters (nursing staff, general practice assistants, patients/next of kin) | Posters (nursing staff, patients/next of kin) | Poster (patients) |
| Information leaflets (GPs, nursing staff, patients/next of kin) | Information leaflets (physicians, nursing staff, patients/next of kin) | Information leaflets (physicians, nursing staff, patients/next of kin) | Information leaflets (patients/next of kin) |
| Presentation for internal education (nursing staff ) | Presentation for internal education (nursing staff) | Presentation for internal education (nursing staff) | Presentation for internal education (nursing staff) |
| Active monitoring checklist (nursing staff) | Active monitoring checklist (nursing staff) | Active monitoring checklist (nursing staff) | Active monitoring checklist (nursing staff) |
|  | E-learning (physicians, nursing staff) | E-learning (physicians, nursing staff) | Information films (physicians, nursing staff, patients/caregivers) |
|  | Case study (physicians, nursing staff) | Case study (physicians, nursing staff) | Case study (physicians, nursing staff) |
|  | Guideline summary Verenso (physicians) | Antibiotic feedback report of the nursing home (physicians, nursing staff) | Swedish guideline summary (physicians) |
|  | Alternative pre-existing pocket card (nursing staff) and information leaflet (patients/next of kin) from guideline | Mobile link to decision-tool (physicians, nursing staff) |  |

**References**

1. van Buul LW, Vreeken HL, Bradley SF, Crnich CJ, Drinka PJ, Geerlings SE, et al. The Development of a Decision Tool for the Empiric Treatment of Suspected Urinary Tract Infection in Frail Older Adults: A Delphi Consensus Procedure. J Am Med Dir Assoc. 2018;19(9):757-64.

2. Hartman EAR, Groen WG, Heltveit-Olsen SR, Lindbaek M, Hoye S, Sundvall PD, et al. Decisions on antibiotic prescribing for suspected urinary tract infections in frail older adults: a qualitative study in four European countries. Age Ageing. 2022;51(6).

3. NORM/NORM-VET. Usage of Antimicrobial Agents and Occurrence of Antimicrobial Resistance in Norway. Tromsø / Oslo; 2020. Contract No.: ISSN:1502-2307 (print) / 1890-9965 (electronic).

4. : Norwegian Organization for Quality Improvement of Laboratory Examinations (Noklus); [Available from: <https://www.noklus.no/en/the-norwegian-organization-for-quality-improvement-of-laboratory-examinations/>.

5. Holecki MD, J; Hryniewicz, W; Imiela, J; Klinger, M; Pawlik, K; Wanke-Ryt, M. Rekomendacje diagnostyki, terapii i profilaktyki zakażeń układu moczowego u dorosłych. Narodowy Program Ochrony Antybiotyków na lata 2011-2015: Narodowy Instytut Leków, Warszawa; 2015. Report No.: 978-83-938000-4-9

6. Richtlijn Urineweginfecties bij kwetsbare ouderen. Dutch guideline. 2018.

7. Bouma M GS, Klinkhamer S, Knottnerus BJ, Platteel TN, Reuland EA, Visser HS, Wolters RJ. NHG-standaard Urineweginfecties. Dutch guideline. 2020.

8. Akselsen PEO, S. Urinveisinfeksjoner i sykehjem, versjon 2.2. Norwegian guideline. 2020.

9. Läkemedelsbehandling av urinvägsinfektioner i öppenvård - behandlingsrekommendation. Swedish guideline. 2017.

10. Molstad S, Lofmark S, Carlin K, Erntell M, Aspevall O, Blad L, et al. Lessons learnt during 20 years of the Swedish strategic programme against antibiotic resistance. Bull World Health Organ. 2017;95(11):764-73.
